# Impact of a pyridazine derivative on tripartite synapse ultrastructure in hippocampus: a three-dimensional analysis

**DOI:** 10.3389/fncel.2023.1229731

**Published:** 2023-08-21

**Authors:** Zan Xu, Joshua B. Foster, Rashelle Lashley, Xueqin Wang, Emily Benson, Grahame Kidd, Chien-liang Glenn Lin

**Affiliations:** ^1^Department of Neuroscience, College of Medicine, The Ohio State University, Columbus, OH, United States; ^2^Department of Neuroscience, Lerner Research Institute, Cleveland Clinic, Cleveland, OH, United States

**Keywords:** tripartite synapse, 3D electron microscopy, pyridazine derivative, spine apparatus, synaptic plasticity, tertiary dendrites

## Abstract

**Introduction:**

We previously discovered a pyridazine derivative compound series that can improve cognitive functions in mouse models of Alzheimer’s disease. One of the advanced compounds from this series, LDN/OSU-0215111-M3, was selected as the preclinical development candidate. This compound activates local protein translation at the perisynaptic astrocytic process (PAP) and enhances synaptic plasticity sequentially. While biochemical evidence supports the hypothesis that the compound enhances the structural plasticity of the tripartite synapse, its direct structural impact has not been investigated.

**Methods:**

Volume electron microscopy was used to study the hippocampal tripartite synapse three-dimensional structure in 3-month-old wild-type FVB/NJ mice after LDN/OSU-0215111-M3 treatment.

**Results:**

LDN/OSU-0215111-M3 increased the size of tertiary apical dendrites, the volume of mushroom spines, the proportion of mushroom spines containing spine apparatus, and alterations in the spine distribution across the surface area of tertiary dendrites. Compound also increased the number of the PAP interacting with the mushroom spines as well as the size of the PAP in contact with the spines. Furthermore, proteomic analysis of the isolated synaptic terminals indicated an increase in dendritic and synaptic proteins as well as suggested a possible involvement of the phospholipase D signaling pathway. To further validate that LDN/OSU-0215111-M3 altered synaptic function, electrophysiological studies showed increased long-term potentiation following compound treatment.

**Discussion:**

This study provides direct evidence that pyridazine derivatives enhance the structural and functional plasticity of the tripartite synapse.

## Introduction

The tripartite synapse consists of a presynaptic terminal, a postsynaptic spine, and a perisynaptic astrocytic process (PAP) that sheaths the synapse, enabling communication between neuron-neuron and neuron-astrocyte (Perea et al., 2009; Lalo et al., 2021). Glutamate is released from the presynaptic terminal and binds to ionotropic or metabotropic receptors on the postsynaptic spine. The PAP regulates glutamate uptake through excitatory amino acid transporters to maintain glutamate homeostasis and prevent excitotoxicity (Lin et al., 2012). Loss of the tripartite synapse is suggested as an underlying mechanism of many neurological disorders, including Alzheimer’s disease (AD) (Sze et al., 1997; Ingelsson et al., 2004; Scheff et al., 2006; Arendt, 2009; Kandimalla et al., 2018; Kashyap et al., 2019). Prevention of loss or enhancement of remaining tripartite synapses may be a potential therapeutic approach for AD and other neurological diseases (Rudy et al., 2015; Takahashi et al., 2015a; Hulshof et al., 2022). We previously discovered a pyridazine-derivative compound series that was found to modulate the tripartite synapse structure and function (Foster et al., 2018). We generated many derivatives of this series (Xing et al., 2011). We have assessed our compounds in AD mouse models (Kong et al., 2014; Takahashi et al., 2015b; Foster et al., 2019), and several other disease models (Kong et al., 2014; Wang et al., 2021). Efficacy studies indicated that our compounds provide a range of benefits to slow the disease progression. Mechanistic studies revealed that our compounds enhance structural and functional plasticity of tripartite synapse via activation of local translation in the PAP (Foster et al., 2018). In mice, at 4-h post compound treatment, a set of proteins increased rapidly in the PAP but not in the cell bodies, main astrocytic processes, or nerve terminals, suggesting that the compound action site is located at the PAP. At 7-day post compound treatment, an increase of synaptic proteins expression and strengthened synaptic long-term potentiation (LTP) were observed. These results suggest that the compound enhances PAP plasticity which consequently regulates synaptic plasticity.

Previously, we found biochemical evidence that supports the notion that our pyridazine compounds may also affect tripartite synapse morphology. The tripartite synapse responds dynamically to environmental stimuli and pathological conditions (Lushnikova et al., 2009; Bellesi et al., 2015; Zhuravleva et al., 2020). However, the light microscope is not sufficient for studying its morphological changes because the PAP, dendritic processes, and axonal processes have diameters below the limit of resolution (Harris and Stevens, 1989; Harris et al., 1992; Aten et al., 2022). To overcome this limitation, super resolution microscopy and electron microscopes can be used to visualize the nanoscale structure of the tripartite synapse. For example, three-dimensional (3D) stochastic optical reconstruction microscopy analysis provides high-resolution imaging down to 10 nm and 3D reconstruction of multiple components within a single synapse (Aleksejenko and Heller, 2021). However, a limitation of this method is that only a small region can be imaged at once, and only structures labeled with fluorescent dyes can be visualized (Bond et al., 2022). Electron microscopy (EM) is the optimal method for visualizing all compartments of the tripartite synapse in the hippocampal CA1 region. Volume EM allows for 3D reconstruction, which is necessary for obtaining measurements such as volume and contact areas to further understand the interactions within the tripartite synapse. Numerous studies have successfully employed 3D EM analysis to investigate the interactive structural relationships of the tripartite synapse under different conditions (Lushnikova et al., 2009; Bellesi et al., 2015; Jawaid et al., 2018).

The purpose of the present study is to determine the morphological changes that occur after treatment with pyridazine-derivatives by using volume EM to reconstruct hippocampal CA1 tripartite synapses. We found that LDN/OSU-0215111-M3 increased the size of tertiary apical dendrites, the volume of mushroom spines, and the proportion of mushroom spines with spine apparatus. LDN/OSU-0215111-M3 also increased the number of PAP in contact with the mushroom spines as well as the branchlet PAPs in contact with the spines. Proteomic analysis of isolated synaptosomes indicated an increase in synaptic and dendritic proteins and suggested that the phospholipase D (PLD) signaling pathway may be involved in this process. Furthermore, electrophysiological study showed increased LTP in compound-treated mice. Overall, this study provides direct evidence that pyridazine derivatives enhance the structural and functional plasticity of the tripartite synapse.

## Materials and methods

### Animals

For the volume EM and mass spectrometry study, we utilized 3-month-old wild-type FVB/NJ mice (Jackson; Stock #001800). For the electrophysiology and biochemical protein expression experiments, male Sprague-Dawley (Charles River, Strain code 001) rats (250–300 g) were used. All animals were housed on a 12-h light/dark cycle with access to food and water *ad libitum*. All experiments were approved by the Institutional Animal Care and Use Committee of The Ohio State University and the National Institutes of Health Guide for the Care and Use of Laboratory Animals.

### Compound and treatment

LDN/OSU-0215111-M3 (hereinafter referred to as M3), which is a metabolite of LDN/OSU-0215111 (Foster et al., 2019), was used in the current study. It is an advanced compound being developed for clinical use, based on the previously characterized pyridazine-derivative LDN/OSU-0212320 (Kong et al., 2014). After completion of this previous study, metabolite profiling studies found that LDN/OSU-0215111 is rapidly (<1 h) and efficiently (>90%) metabolized into M3 in rodents. M3 was chosen as it has higher potency than previous derivatives and its’ metabolite profile is consistent between species unlike the parent compound. The purify of M3 was >95% and is highly soluble in water (>100 mg/mL). Mice were treated daily for 7 days via voluntary oral administration with vehicle or M3 (40 mg/kg). M3 was dissolved in ddH_2_O (0.1 mg/μL), diluted with 50% honey (50% ddH_2_O), and dosed orally to individual animals using a pipette based on body weight (final volume < 100 μL). The mice were trained for 3 days to voluntarily consume M3 by limiting food intake to 85% of body weight. Rats were treated daily for 7 days with vehicle (saline) or M3 at respective dose for each experiment (final volume = 1,000 μL). Rats were scruffed and treated by oral gavage.

### Serial block face scanning EM (SBF-SEM)

After 7-day treatment, mice were perfused with 2.5% glutaraldehyde and 4% paraformaldehyde. Brains were removed and sliced coronally using a 0.5 mm mouse brain matrix (Electron Microscopy Sciences). Slices containing the CA1 region were sequentially treated with osmium ferricyanide, thiocarbohydrazide, aqueous osmium (2%), aqueous uranyl acetate, and Walton’s lead aspartate stain, dehydrated and embedded in Epon (all reagents from Electron Microscopy Sciences, Hatfield, PA). The plastic embedded CA1 hippocampal sections were trimmed, mounted on pins, and imaged in a Carl Zeiss Sigma VP scanning electron microscope equipped with a 3View2 in-chamber ultramicrotome system (Gatan, Warrendale, PA, USA) and a high sensitivity, low-kV backscattered electron detector (Gatan, Warrendale, PA, USA). Sets of 300–500 images were serially collected at 5–7 nm per pixel resolution, 48 μm × 48 μm size and at 75 nm thickness (2.2 kV, 30 μm aperture) using SBF-SEM.

### 3D reconstruction and analysis

Four dendrites and their spines for each animal (*n* = 3 animals) were traced (*n* = 12 traces/group) and reconstructed in 3D using Reconstruct software (Fiala, 2005 JMicrosc). The spines were categorized by their shapes based on the previous established criteria: (1) mushroom: spine head diameter is at least two times the spine neck diameter; (2) stubby: short spines (<1 μm) with spine neck diameter similar to spine head diameter; (3) thin: neck diameter less than 0.2 μm and the spine head diameter is less than two times the neck diameter (Jawaid et al., 2018). The volume of the spines was calculated by Reconstruct. The PAPs were identified by ultrastructural characteristics, including the presence of glycogen granule and the acute angles of membranes associated with other cells (most cells have rounded and obtuse angle processes). Where the ultrastructural characteristics of individual astrocyte processes were unclear, the process was followed through adjacent EM slices to a main astrocyte trunk containing glycogen and/or characteristic glial filament bundles. The presence or absence of PAPs was investigated at each spine. The PAPs were designated as cleft-associated PAPs (contacting both presynaptic terminal and post synaptic spine) and non-cleft PAPs (making contact with either the presynaptic terminal or the post synaptic spine). Each cleft-associated PAP was further classified as a leaflet, branchlet, or branch based on the diameter of the PAP adjacent to the synapse (leaflet: <250 nm, branchlet: 250 nm–800 nm, branch: >800 nm) (Aboufares El Alaoui et al., 2021).

### Synaptosome isolation

The procedure for isolating synaptosomes was performed as previously described (Foster et al., 2018). In brief, brains were collected from mice and the forebrain was dissected out. Forebrains were then suspended in 10 × volume of homogenization buffer (sucrose-EDTA buffer, 0.25 mM DTT and 1 × protease inhibitor) and homogenized. The homogenate was then centrifuged at 1,000 × *g* for 10 min. A Percoll (Sigma-Aldrich, St. Louis, MO, USA) gradient (2, 6, 10, and 20%) was then used to separate different types of vesicles. We collected the F3 (between 6–10%; mixed PAPs and synaptosomes) or F4 (between 10–20%; synaptosomes) fractions resuspended in in 1 × PBS with 1 × protease inhibitor for Western blotting and proteomic analysis.

### Proteomic analysis

Experimental procedures were performed as previously indicated (Foster et al., 2018). Proteomic data was generated by the Proteomics Shared Resource Core at The Ohio State University, using capillary-LC/MS/MS on a Thermo Scientific Orbitrap Fusion mass spectrometer equipped with a nanospray FAIMS Pro™ Source. MS/MS data was searched using Mascot Daemon by Matrix Science version 2.7.0 via Proteome Discoverer (version 2.4 Thermo Scientific), against the most recent Uniprot databases. Data was filtered at 1% FDR and proteins identified with at least two unique peptides were considered reliable. Label-free quantitation was performed using the spectral count approach, and Scaffold (Proteome Software, Portland, OR, USA) was used for data analysis. Student-t tests were performed to evaluate significant fold changes (*p* < 0.05).

### Western blotting

Western blotting was performed as described previously with the following changes (Foster et al., 2018). Normalized amounts of protein were separated by SDS-PAGE (8% polyacrylamide gel) and transferred onto nitrocellulose membranes. The membranes were then probed with specific antibodies, including excitatory amino acid transporter 2 (EAAT2) (Guo, 2003), post-synaptic density 95 (PSD-95, Thermo Fisher, AB_2092361), and synaptic vesicle glycoprotein 2A (SV2A, Abcam, AB_778192). Subsequently, the blot was incubated with goat anti-rabbit IgG or anti-mouse IgG secondary antibodies (Bio-Rad; 1:5,000) conjugated to horseradish-peroxidase. Immuno-reactive bands were visualized using enhanced chemiluminescence substrates according to the manufacturer’s instructions. Digital images of the immunoblots were captured using the ChemiDoc Imaging System (Bio-Rad), and band intensities were analyzed with Image Lab (Bio-Rad). Data is reported as fold change relative to the appropriate vehicle sample.

### Functional GO enrichment analysis

The proteins that showed an increase of over 1.5-fold after M3 treatment were submitted on Metascape^1^ for partitioning. Metascape identified overexpression gene ontology (GO) categories and protein-protein interactions (*p* < 0.01). The enrichment analysis covered biological processes (BP), cellular components (CC), molecular functions (MF), and Kyoto Encyclopedia of Genes and Genomes (KEGG) pathway.

### fEPSP recordings

The experimental procedures were conducted following previous indications but with some modifications (Foster et al., 2018). The tissue assessment was done after 7 days of treatment, precisely 24 h after the dose on day 7. Following anesthesia, the head was immediately decapitated, and the brain was rapidly removed and placed in ice-cold cutting solution. Hippocampal coronal slices (400 μm) were prepared and transferred to a chamber containing artificial cerebrospinal fluid (aCSF) bubbled with 95% O_2_ and 5% CO_2_ at 37°C for 30 min before being transferred to a room temperature solution for at least 1 h. The local Field excitatory postsynaptic potentials (fEPSPs) were recorded from the stratum radiatum of the dorsal CA1 hippocampal area and evoked by electrical stimulation (100 μs duration, every 30 s) of the pathway of Schaffer collateral fibers (SC-CA1). Input-output (I/O) curves were generated for each slice with stimulation intensity varying from 0 to 0.5 mA, in steps of 0.05 mA. Based on the I/O curves, the stimulation intensity that evokes approximately 50% of the maximum response was chosen for the subsequent experiment. LTP was induced with theta-burst stimulation and the peak of the evoked fEPSPs was measured and normalized to the preconditioning 5-min averaged baseline.

### Statistical analysis

Statistical analyses were conducted with GraphPad Prism 5.0 (GraphPad Software, Inc., La Jolla, CA). All results are presented as mean ± standard deviation except where noted. The Shapiro–Wilk test was used to test for normal distribution. Comparisons were made by Student’s two-tailed unpaired *t*-tests incorporating Bonferroni’s corrections for multiple comparisons where appropriate and using F-tests for variance analysis. Chi-square tests were conducted for small, medium, large spine comparison and leaflet, branchlet, and branch PAP comparisons. Linear regression analysis was used to assess the relationship between spine density and dendrite diameter and the quantification of the tripartite synapse proteins quantified via Western blot. The I/O curve stimulation values were log_10_ transformed. The I/O curve was further assessed by linear regression analysis. A Student’s *t*-test was used to assess the last 5 min of each LTP plot. Here, each time point from 35 min to 40 min for each treatment group was averaged together for this analysis. Significance was set as *p* < 0.05.

## Results

**FIGURE 1:**
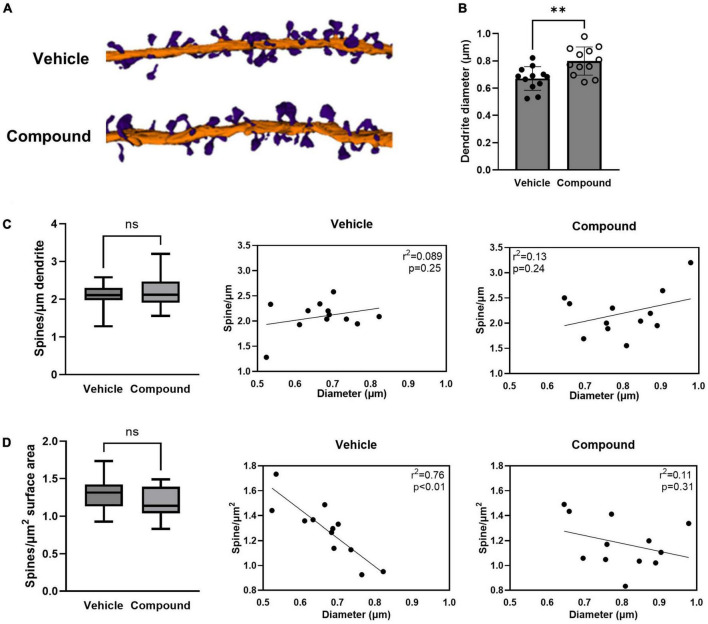
LDN/OSU-0215111-M3 increased the diameter of the tertiary apical dendrite and altered the dynamic of the dendritic spine distribution. Mice were treated with M3 for 7 consecutive days and neurons in hippocampal CA1 stratum radiatum layers were subjected to SBF-SEM. **(A)** Representative images of 3D reconstructions of the tertiary apical dendrites with dendritic spines (Purple = spines; Orange = dendritic shaft). **(B)** The dendritic shaft diameter increased following M3 treatment [*t*(22) = 3.28; *p* < 0.01]. **(C)** The number of spines/μm of dendrite was unchanged by M3 treatment [*t*(22) = 0.65; *p* = 0.53, left]. Scatter plots showing the very weak positive correlation between the diameter of the dendritic shaft and the spines/μm in vehicle (middle) and M3 (right) treated mice. **(D)** The number of spines/μm^2^ of dendrite was similar between vehicle (1.3/μm^2^) and M3 [1.2/μm^2^; *t*(22) = 0.22; *p* = 0.21, left]. Scatter plots showing the relation between the diameter of the dendritic shaft and spines/μm^2^ in vehicle (middle) and M3 (right) treated mice. M3 treatment decoupled the correlation of surface density of spines to dendrite diameter that was observed in the vehicle group. Values presented as mean ± SD. *n* = 12 (3 animals and 4 dendrites/animal) per group. ^**^p < 0.01. ns, not significant.

We used volume EM to elucidate the 3D ultrastructural changes of the synaptic spines and the surrounding astrocytic processes in the mouse brain after LDN/OSU-0215111-M3 treatment. Mice were treated with vehicle or M3 at 40 mg/kg/day (*n* = 3 each) for 7 consecutive days before being perfused for serial block face scanning electron microscope (SBF-SEM). We performed 3D reconstruction on 4 dendrites from each animal’s hippocampus CA1 stratum radiatum. The tertiary dendrites, which were cut longitudinally during the SBF-SEM imaging process, were selected and traced from each animal. The tracing of a dendrite stopped when the longitudinal cut became a cross-sectional cut. The length of the traced dendrites ranged from 10 to 41 μm. A total of 12 dendrites from vehicle-treated mice and 12 dendrites from M3-treated mice were reconstructed. All spines extending from the reconstructed dendrites were traced. A total of 379 spines for vehicle-treated mice and 582 spines for M3-treated mice were reconstructed. Figure 1A shows representative dendrites from vehicle- and M3-treated mice.

### LDN/OSU-0215111-M3 increases the size of the tertiary dendritic shafts and alters the spine distribution, but not density

We first investigated if LDN/OSU-0215111-M3 affected the size of the dendritic shaft and the spine density and distribution. We compared the diameter of the dendrites between vehicle- and M3-treated mice. Analysis of reconstructed dendrites showed a significant increase in the mean diameter of the dendritic shaft in M3 (0.80 μm) compared to vehicle (0.67 μm) treated mice (Figure 1B). We then assessed the spine density by calculating the number of spines per micrometer of dendrite length. As shown in Figure 1C, left panel, both vehicle- and M3-treated mice had an equivalent average density of spines (2.1/μm). When plotted, there was little relationship between dendrite diameter and spine density for both vehicle and M3; most dendrites had ∼1.5–2.5 spines per μm (Figure 1C, middle and left panels). To assess whether the M3 affected the relationship between spine number and dendritic surface area, we compared the spine number per unit surface area between vehicle- and M3-treated mice. As shown in Figure 1D, left panel, the mean spine number/μm^2^ was similar at 1.2/μm^2^ for both vehicle- and M3-treated mice. However, an insignificant trend toward fewer spines per unit surface area in M3-treated mice was suggested by the different medians (*p* = 0.24). To investigate further, we plotted dendritic shaft diameter and spine number/μm^2^. In vehicle-treated mice, spine number/μm^2^ had a strong negative correlation with the dendrite shaft diameter (Figure 1D, middle panel; *R*^2^ = 0.76, *p* < 0.01). Following M3 treatment, the spine number/μm^2^ became decoupled from the size of the dendritic shaft diameter (Figure 1D, right panel; *R*^2^ = 0.11, *p* = 0.31). Overall, these results indicate that M3 treatment increases the size of the tertiary dendrites and alters spine distribution, but not spine density along the tertiary dendrites.

### LDN/OSU-0215111-M3 does not change the proportion of each mushroom, thin, and stubby spine type but alter their distribution and increase the size of the mushroom spines

**FIGURE 2:**
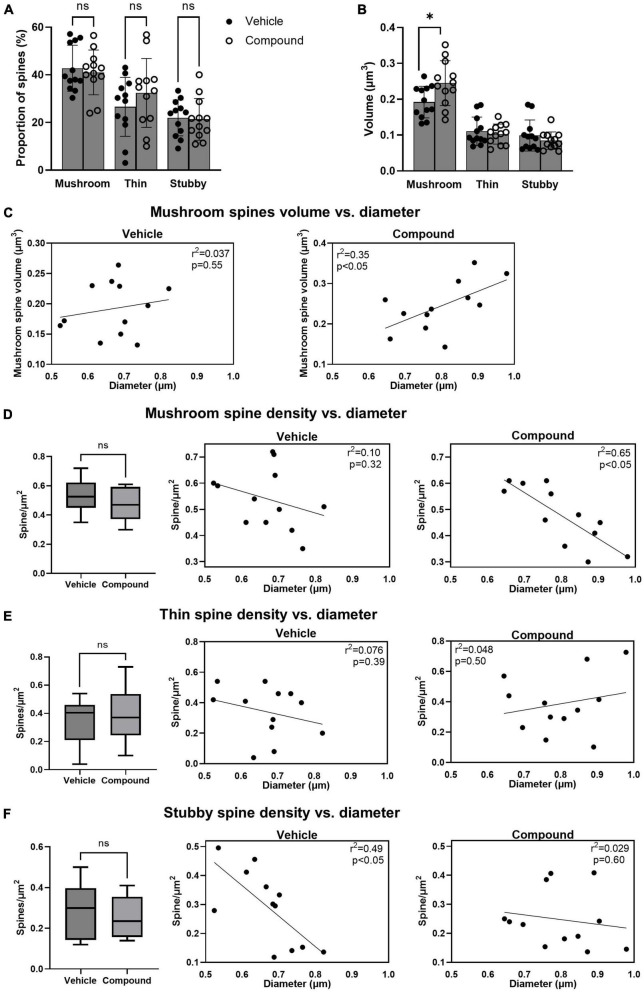
LDN/OSU-0215111-M3 did not change the proportion of each spine type but altered their distribution and increased the volume of the mushroom spines. Spines were categorized into mushroom, thin and stubby and compared between vehicle and M3. **(A)** The proportion of each type of spine was similar between vehicle and M3 [mushroom: *t*(22) = 0.44, *p* = 0.86; Thin: *t*(22) = 1.06, *p* = 0.81; Stubby: *t*(22) = 0.18, *p* = 0.83]. **(B)** The mean volume of the mushroom spines was significantly increased following M3 treatment (0.24 μm^3^) compared to vehicle [0.19 μm^3^; *t*(22) = 2.38, *p* < 0.05] while the mean volume of the thin and stubby spines were similar between vehicle and M3 [Thin: *t*(22) = 0.54, *p* = 0.54; Stubby *t*(22) = 0.99, *p* = 0.34]. **(C)** Scatter plots showing a positive relation between the diameter of the dendritic shaft and mushroom spines volume in vehicle (left) and M3 (right) treated mice. M3 treatment significantly improved the correlation. The number of mushroom, thin and stubby spines/μm^2^ were compared between vehicle and M3. **(D)** The number of mushroom spines/μm^2^ were similar between vehicle (0.54/μm^2^) and M3 treatment [0.48/μm^2^; *t*(22) = 1.33; *p* = 0.11] (left panel). Scatter plots showed a non-linear relationship between the diameter of the dendritic shaft and mushroom spines/μm^2^ in vehicle treated mice (middle panel) while M3 treatment established a strong negative correlation between dendrite diameter and mushroom spines/μm^2^ (right panel). **(E)** The number of thin spines/μm^2^ were similar between vehicle (0.34/μm^2^) and M3 treatment [0.39/μm^2^; *t*(22) = 0.63; *p* = 0.53] (left panel). Scatter plots show a non-linear relationship between the diameter of the dendritic shaft and thin spines/μm^2^ in vehicle treated mice (middle panel) and M3 treated mice (right panel). **(F)** The number of stubby spines/μm^2^ were similar between vehicle (0.29/μm^2^) and M3 treatment [0.25/μm^2^; *t*(22) = 0.89; *p* = 0.38] (left panel). Scatter plots show a linear relationship between the diameter of the dendritic shaft and stubby spines/μm^2^ in vehicle treated mice (middle panel) which is lost after M3 treatment (right panel). Values presented as mean ± SD. *n* = 12 (3 animals and 4 dendrites/animal) per group. ns, not significant. *p < 0.05.

Next, we categorized the spines as mushroom, thin, and stubby to investigate whether M3 affected the number, the size, and the distribution of each spine type. Results showed that the percentage of the total number of mushroom, thin, and stubby spines was similar in vehicle- and M3-treated mice (Figure 2A). However, the mean volume of the mushroom spines significantly increased following M3 treatment (0.24 μm^3^) compared to vehicle (0.19 μm^3^) while the mean volume of the thin and stubby spines was unchanged (Figure 2B). We then asked whether larger dendrites had greater mushroom spine volume. In vehicle-treated mice, larger volume spines did not correlate with dendrite diameter (Figure 2C, left panel, *R*^2^ = 0.037, *p* = 0.55). However, after treatment, there was a strong correlation where the larger diameter dendrites had the greater volume mushroom spines (Figure 2C, right panel, *R*^2^ = 0.35, *p* < 0.05). Next, we investigated whether dendrite diameter was associated with the number of mushroom spines. Results showed that the mean density of mushroom spine number/μm^2^ was similar between vehicle and M3 treatment (Figure 2D, left panel). However, the number of mushroom spine number/μm^2^ of the dendrite had a stronger negative correlation with the diameter of the dendritic shaft following M3 treatment (*R*^2^ = 0.65, *p* < 0.05) compared to the vehicle (*R*^2^ = 0.10, *p* = 0.32; Figure 2D, middle and right panels). We also examined the distribution of thin spines and stubby spines. The mean densities (spine number/μm^2^) for both thin spines and stubby spines were similar between vehicle and M3 treatment (Figures 2E, F, left panels). The number of thin spine/μm^2^ of the dendrite had a very weak correlation with the dendrite shaft diameter independent of treatment (Vehicle: *R*^2^ = 0.076, *p* = 0.39; M3: *R*^2^ = 0.048, *p* = 0.50; Figure 2E, middle and right panels). The stubby spine number/μm^2^ of the dendrite had a strong negative correlation with the diameter of the dendrite shaft in the vehicle treated mice (*R*^2^ = 0.49, *p* < 0.05) which was lost in the M3 treated mice (*R*^2^ = 0.029, *p* = 0.60; Figure 2F, middle and right panels). Together, these data indicates that M3 does not change the proportion of each spine type but increases the size of the mushroom spines and strengthens the association between mushroom spine volume and dendritic shaft diameter. M3 strongly stabilizes the dynamic distribution of mushroom spines across the dendritic shaft and alters how thin and stubby spines are distributed. These results also explain the reason for the lack of correlation between spine density per surface area and the diameter of the dendritic shaft following M3 treatment (the result of Figure 1D).

### LDN/OSU-0215111-M3 increases the number of mushroom spines containing spine apparatus

**FIGURE 3:**
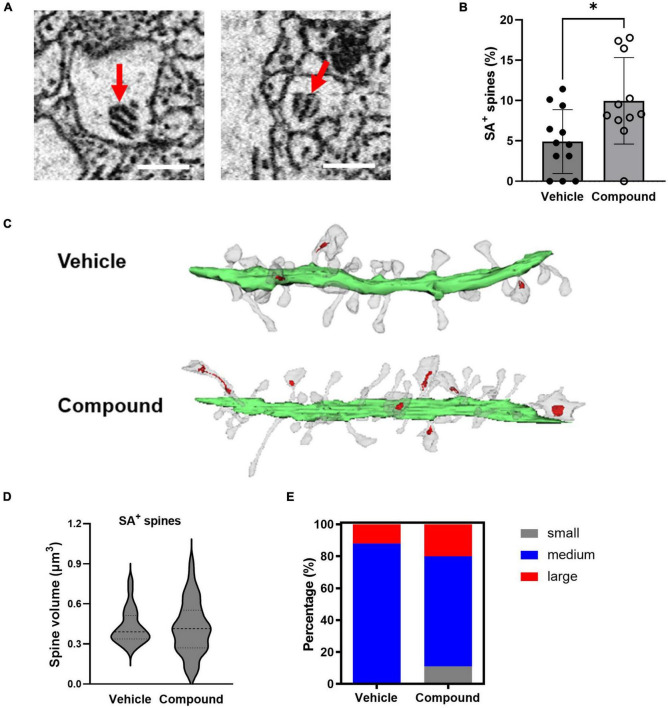
LDN/OSU-0215111-M3 increased the number of mushroom spines containing SA and the proportion of small and large SA^+^ mushroom spines. **(A)** Representative images of SA from our EM images (scale bar = 0.5 μm). **(B)** The proportion of SA^+^ spines increased following M3 treatment (4.92%) compared to vehicle (9.97%) [*t*(21) = 2.59; *p* < 0.01]. **(C)** Representative images of dendrites (Green = dendritic shafts; Grey = dendritic spines; Red = SA). **(D)** The mean SA^+^ spines size is similar between vehicle (0.43 μm^3^) and M3 (0.42 μm^3^) [*t*(78) = 0.21; *p* = 0.81]; The variance of the size of the SA^+^ spines were significantly different between and vehicle and M3 (*F* = 2.31; *p* < 0.05). **(E)** The percentage of small (0–0.2 μm^3^), medium (0.2–0.6 μm^3^), and large (0.6–0.8 μm^3^) SA^+^ spines were significantly different following M3 treatment compared to vehicle (X ^2^ (2, *N* = 200) = 15.3, *p* < 0.001). Values presented as mean ± SD, *n* = 12 (3 animals and 4 dendrites/animal) per group. *p < 0.05.

The spine apparatus (SA) is a specialized form of smooth endoplasmic reticulum and is associated with mature and mushroom spines (Segal et al., 2010). Figure 3A shows representative images of SA from two spines. To investigate if M3 affected the distribution of SA, we calculated the percentage of mushroom spines with SA (SA^+^ spines) in relation to the total number of the spines from each dendrite. Results showed that M3 significantly increased the percentage of SA^+^ spines (Figure 3B). Figure 3C shows representative dendrites with SA^+^ spines (indicated in red) from vehicle- and M3-treated mice. The average volume of SA^+^ spines for both vehicle and M3 treated mice was approximately 0.4 μm^3^ (Figure 3D). While M3 did not change the overall average SA^+^ spine volume, M3 significantly increased the variance of SA^+^ spine size (*F* = 2.30, *p* = 0.025; Figure 3D). To assess this change in SA^+^ spine size variance, the SA^+^ spines were further separated into small (0–0.2 μm^3^), medium (0.2–0.6 μm^3^), and large (0.6–0.8 μm^3^) size categories. The percentage of small, medium, and large SA^+^ spines was significantly different following M3 treatment [X ^2^ (2, *N* = 200) = 15.3, *p* < 0.001; Figure 3E]. Specifically, M3 treatment increased the percentage of small and large spines containing SA^+^ while concurrently decreasing the percentage of medium SA^+^ spines. These results indicate that M3 increases the number of mushroom spines containing SA as well as the proportion of small and large mushroom spines that contain SA.

### LDN/OSU-0215111-M3 increases the size of PAP in contact with the spines and the number of PAP interacting with mushroom spines

**FIGURE 4:**
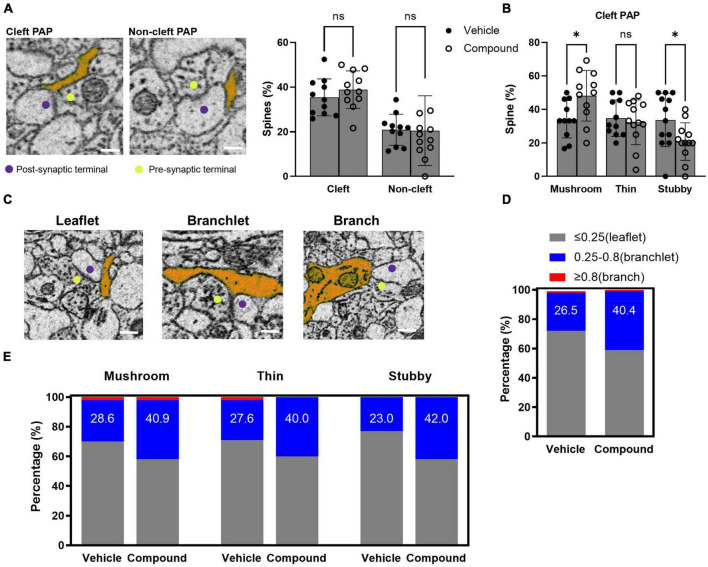
LDN/OSU-0215111-M3 increased the PAP contact with mushroom spines due to an increased proportion of branchlet PAPs at the synapse. **(A)** Left panel: representative images for cleft-associated PAP and non-cleft PAP (scale bar = 0.5 μm); Right panel: The percentage of spines with cleft-associated PAP was similar between vehicle (35.4%) and M3 (38.9%) treated mice [*t*(20) = 0.98; *p* = 0.67]. **(B)** The percentage of mushroom spines with cleft-associated PAP was increased following M3 (48.2%) treatment compared to vehicle (34.2%) while thin spines were similar (32.4 and 34.8%, respectively) and stubby spines decreased following M3 treatment [33.7 to 20.9%, respectively; mushroom: *t*(20) = 2.49, *p* < 0.05; Thin: *t*(21) = 0.48, *p* = 0.84; Stubby: *t*(22) = 2.29, *p* < 0.05]. **(C)** The representative images for leaflet, branchlet, and branch PAP (scale bar = 0.5 μm). **(D)** The percentage of spines with branchlet PAP increased following M3 (40.4%) treatment compared to vehicle [26.5%; X ^2^ (1, *N* = 197) = 4.26, *p* < 0.05]. **(E)** The percentage of stubby spines with branchlet PAP significantly increased following M3 (42.0%) treatment compared to vehicle (23.0%) [X ^2^ (1, *N* = 200) = 8.23, *p* < 0.01]. The percentage of mushroom and thin spines with branchlet PAP increased but not significantly. ns, not significant. *p < 0.05.

Perisynaptic astrocytic processes are highly dynamic and regulate synaptic activity. In our previous studies, we found that M3 treatment results in rapid up-regulation of a set of proteins in the PAP via activation of local translation in PAP (Foster et al., 2018). Here, we analyzed the morphology of the PAPs contacting the synapse following M3 treatment. We classified these PAPs based on their interaction with the synapses. A cleft-associated PAP was defined by a PAP contacting both presynaptic terminal and post-synaptic spines, and a non-cleft PAP described a PAP contacting either presynaptic terminal or post synaptic spine (Figure 4A, left panel). The presynaptic terminals that synapsed on the traced spines were identified by the presence of synaptic vesicles and the release site between the synapses. We determined the percentage of spines that had cleft-associated PAP or non-cleft PAP. Results from both control and treated samples showed that around 35% of the spines were in contact with cleft-associated PAP, and 20% of the spines were in contact with non-cleft PAP (Figure 4A, right panel). The proportion of the spines with cleft-associated PAP was similar for vehicle (35.4%) and M3 (38.9%) treated mice (Figure 4A, right panel). Next, we assessed the percentage of each type of spines with PAP contact. Results showed that the percentage of mushroom spine with cleft-associated PAP contact increased following M3 (48.2%) treatment compared to vehicle (34.2%), and the percentage of stubby spines with cleft-associated PAP contact decreased following M3 (20.9%) treatment compared to vehicle (33.8%) (Figure 4B). As defined previously (Aboufares El Alaoui et al., 2021), the PAPs were categorized based on their width into leaflet (<0.25 μm), branchlet (0.25–0.8 μm), and branch (>0.8 μm; Figure 4C). Figure 4D shows that the number of branchlet PAPs increased and the number of leaflet PAPs decreased following M3 treatment [X ^2^ (1, *N* = 197) = 4.26, *p* < 0.05]. The number of branchlet PAP increased overall (∼1.5 fold) in all types of spines following M3 treatment (Figure 4E). However, only the stubby spines had a significantly increased percentage of branchlet PAP interaction [X ^2^ (1, *N* = 200) = 8.23, *p* < 0.01; Figure 4E]. These data indicate M3 increases the number of cleft-associated PAPs interacting with the mushroom spine population. The percentage of branchlet PAPs increased in all types of spines. Since there was not a proportionate change in the total number of PAPs, this suggests M3 mediates a conversion of leaflet PAPs to larger branchlet PAPs.

### LDN/OSU-0215111-M3 increases the expression of synaptic and dendritic proteins

**FIGURE 5:**
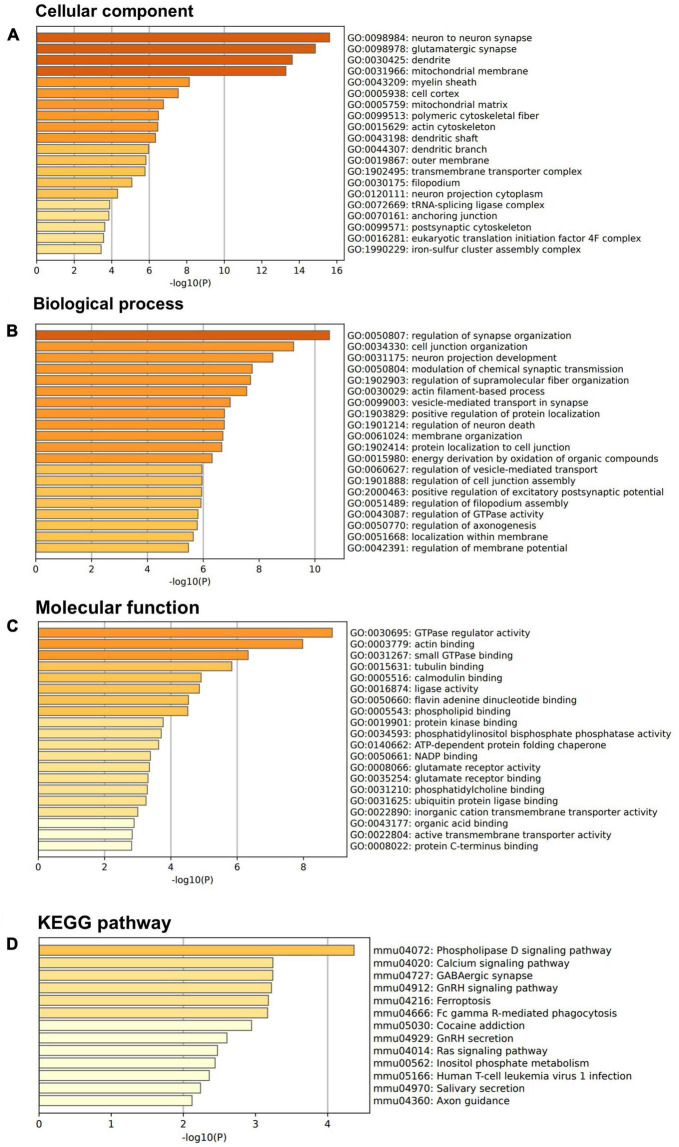
LDN/OSU-0215111-M3 increases the expression of synaptic and dendritic proteins. Isolated synaptosomes from 7-day vehicle or M3 treated mice were subjected to proteomic analysis. A total of 300 proteins were significantly increased 1.5-fold following M3 treatment and were subjected to GO enrichment analysis. **(A)** Bar graphs showed the top 20 terms for GO cellular components terms. **(B)** Bar graphs showed the top 20 terms for GO biological process terms. **(C)** Bar graphs showed the top 20 terms for GO molecular function terms. **(D)** Proteins from the top 3 GO cellular components terms were subjected to KEGG pathway analysis.

Above SBF-EM studies indicated that M3 enhances structure of dendrite and mushroom spines. To further investigate these findings at the molecular level, we used a proteomic approach to determine protein composition of the isolated synaptic terminals (synaptosomes) from the forebrain of mice treated with M3 compared to the mice treated with vehicle. Mice were treated with vehicle or M3 for 7 days, and the forebrains were processed for synaptosome isolation. Isolated synaptosomes were subjected to mass spectrometry. A total of 3,763 proteins were identified from 4 replicates. We identified 300 proteins significantly increased ≥1.5-folds following M3 treatment. We then conducted Gene Ontology (GO) enrichment analysis of these 300 up-regulated proteins. Figure 5 shows the top 20 terms in the bar plot for GO cellular components, GO biological process, and GO biological process terms. To investigate the potential pathway activated by M3 leading to changes observed in the dendrites, we submitted all the proteins belonging to the top three GO terms, neuron to neuron synapse, glutamatergic synapse, and dendrite for KEGG pathway analysis (Figure 5D). The KEGG pathway analysis showed that the top enriched pathway was the phospholipase D (PLD) signaling pathway. The PLD signaling pathway has been implicated in the regulation of dendrites and dendritic spine morphology. These results suggested that M3 may strengthen the dendritic shaft and synapses through activation of PLD signaling pathway.

### LDN/OSU-0215111-M3 enhances synaptic plasticity in the hippocampus

**FIGURE 6:**
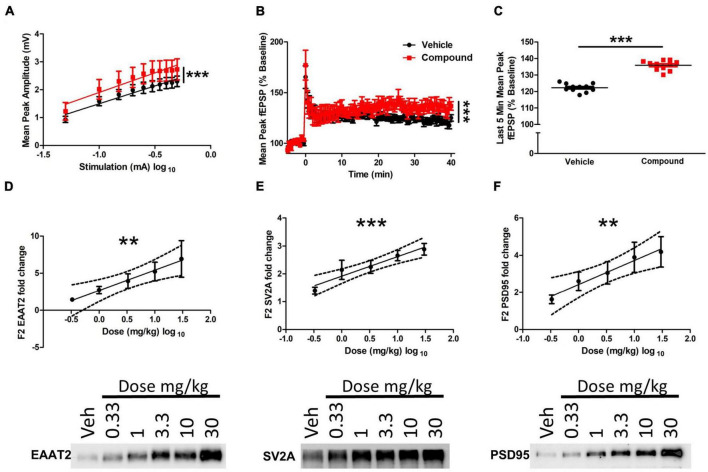
LDN/OSU-0215111-M3 enhanced hippocampal function in Sprague-Dawley rats. **(A)** There was no statistical difference in the slopes of the input-output curves generated from the slices used to record LTP indicating that responses in each group are linear and thus comparable. However, the elevation of the M3 slope was significantly higher indicating an overall stronger synaptic response in the M3 treatment condition compared to the same stimulation as vehicle treated (vehicle: *n* = 4 animals/12 slices; M3: *n* = 4 animals/10 slices). **(B)** Mean long-term potentiation recording traces. Baseline recordings were generated for 5 min before LTP induction. Theta-burst stimulation was used to induce LTP at time point 0. LTP was recorded for 40 min after induction. Linear regression analysis indicates that the slopes of the 2 curves are significantly different over the last 40 min of the recording after LTP induction. **(C)** Importantly, once stable LTP was established, average peak amplitude across all combined time points of the last 5 min of LTP recording were significantly higher in the M3 treated group. The expression level of **(D)** EAAT2, **(E)** SV2A, and **(F)** PSD95 increased following M3 treatment (dose was log transformed) in a dose dependent manner according to linear regression analysis (*n* = 9 animals per dose). Representative blots are shown below for each protein assessed. Dashed lines represent 95% confidence interval for best fit line of linear regression. Data represented as mean ± SEM. **p < 0.01, ***p < 0.001.

To determine the functional consequences of the morphology changes at the dendritic and tripartite synapse level, we used an electrophysiological approach to assess synaptic activity following M3 treatment. Wild-type Sprague-Dawley rats were treated with vehicle or M3 (30 mg/kg) daily for 7 days, and acute hippocampal slices (vehicle = 4 animals/12 slices; M3 = 4 animals/10 slices) were collected and assessed for long-term potentiation (LTP). Input/output curves showed no difference in the slope of the I/O curves suggesting that both treatment conditions exhibited comparable, equally-scaled responses to evoked stimulation at baseline (Figure 6A). However, the elevation of the M3 slope was significantly higher indicating an overall stronger synaptic response in the M3 treatment condition compared to the same stimulation as vehicle treated. Overall, though, synaptic responses in each group were of equal slope and thus comparable. As shown in Figure 6B, field potential recording from CA1 of M3-treated mice showed significantly increased responses to stimulation of CA3 afferents up to 40 min after LTP induction compared to vehicle-treated animals. Once stable LTP was established, the last 5 min of recording were binned together for analysis, which showed that M3 treatment significantly increased the mean peak fEPSP response to equivalent stimulation in vehicles (Figure 6C). These results indicate that M3 treatment enhances synaptic response to stimulation.

Furthermore, to assess M3 effects on tripartite synapse protein expression, rat forebrain was processed through a Percoll gradient to isolate a fraction (F3) of mixed PAP and neuronal processes. Excitatory amino acid transporter 2 (EAAT2) was used as a marker for PAP function as EAAT2 provides the essential function of glutamate uptake at the tripartite synapse. Synaptic vesicle glycoprotein 2A (SV2A) was used as a pre-synaptic bouton marker as SV2A is associated with neurotransmitter release from the pre-synapse. Postsynaptic density protein 95 (PSD95) was also assessed as a proxy for post-synaptic strength. Linear regression analysis of Western blotting showed a strong, dose-dependent increase of EAAT2 (*R*^2^ = 0.19, *p* < 0.01), SV2A (*R*2 = 0.34, *p* < 0.0001), and PSD95 (*R*^2^ = 0.19, *p* < 0.01; Figures 6D–F). Overall, M3 mediates a dose-dependent increase in EAAT2, SV2A and PSD95 proteins which further corroborates the structural changes observed and enhanced LTP response.

## Discussion

In this study, we investigated the effects of a novel, pyridazine derivative compound, LDN/OSU-0215111-M3, on the tripartite synapse in the hippocampus. We employed SBF-SEM to examine M3 mediated effects on the tertiary apical dendrite and the tripartite synapse at the hippocampus CA1 region. Furthermore, we used proteomic analysis to investigate the M3 mediated molecular changes at the synapse and employed electrophysiological techniques to study the resulting effect on synaptic function. Our results showed that M3 increased the diameter of the tertiary apical dendrites, the volume of mushroom spines, and the proportion of SA^+^ mushroom spines. M3 also increased the number of branchlet PAPs in contact with the synapse and altered the dynamics of spine clustering along the length of the dendrite. Proteomic analysis indicated M3 increased the expression of synaptic and dendritic proteins, and electrophysiological studies showed enhanced LTP. Together, M3 mediated molecular, structural, and functional changes resulted in enhanced synaptic plasticity.

We observed that M3 increased the diameter of the tertiary apical dendrite. The size of the dendritic shaft is important for neuronal function and communication. A larger dendritic shaft generally provides a larger surface area for receiving inputs from other neurons, and increases the number of potential synapses (Stuart et al., 2016). Even though the total number of spines was not affected by M3, the distribution of the spines along the dendrites was significantly altered. In vehicle-treated mice, the density of spines along the dendrites exhibited a negative correlation to the diameter of the dendrites. This indicates that the wider dendrites with a larger surface area accommodate a similar number of synapses as narrower dendrites even with a smaller surface area. Thus, the narrow dendrites tend to have a higher density of spines to compensate for the reduced surface area to maintain equivalent total number of spines. This represents a novel finding regarding the distribution of spine density along tertiary apical CA1 dendrites, as previous studies have only looked at the density of spines across the entire population of dendrites, including primary and secondary dendrites originating from the neuron soma (Harris et al., 1992; Trommald et al., 1995; Megías et al., 2001). These studies concluded that the spine density decreases with increasing distance from the soma. Our results, however, indicate that treatment with M3 decoupled this relationship resulting in equal density of spines independent of dendrite diameter in tertiary apical dendrites. Specifically, the larger dendrites exhibited increased surface area spine density after M3 treatment, equivalent to smaller dendrites. Further analysis revealed that the density of stubby spines may account for this change in spine distribution as stubby spines became decoupled from dendritic diameter after M3 treatment in a fashion reminiscent of total spines. Interestingly, however, mushroom spine density, in relation to dendritic diameter, became tightly coupled (negatively correlated) after M3 treatment unlike in the vehicle condition.

Proteomic analysis of M3 treated synaptosomes indicated PLD signaling pathway is the most significantly involved pathway. The PLD signaling pathway is involved in calcium regulation (Bader and Vitale, 2009). Activation of PLD can lead to an increase in intracellular calcium levels which can then trigger a cascade of signaling pathways that regulate various cellular processes (English et al., 1996). Specifically, the PLD signaling pathway is known to play a role in regulating dendrite size and branching in certain neuronal populations (Trommald et al., 1995; Megías et al., 2001; Li et al., 2019). Studies in cultured neurons have shown that PLD activation can increase dendrite size and promote the formation of new branches, possibly by regulating intracellular proteins involved in dendrite growth, such as members of the Rho family of small GTPases (Klein, 2005; Park and Han, 2018). Proteomic analysis of M3 treated synaptosomes further supports this notion as the top GO molecular function term is GTPase regulator activity. PLD signaling can also modulate the activity and localization of enzymes involved in the synthesis and degradation of cytoskeletal components like actin and microtubules which are important for regulating dendrite structure (Klein, 2005; Park and Han, 2018). The electrophysiological data further supported this idea by showing increased LTP, which indicates an increase in the strength of the synaptic connections between neurons following M3 treatment. Therefore, M3 mediated dendritic structural changes may have occurred through a PLD-dependent pathway.

After treatment with M3, we found that the size of the mushroom spines was significantly increased. However, the total number of mushroom spines remained constant. An increase in the size of the spine head is positively correlated with the surface area of the postsynaptic density, the number of postsynaptic receptors, and the amount of neurotransmitter available for release (Harris and Stevens, 1989; Stuart et al., 2016; Gipson and Olive, 2017; Sun et al., 2021). In corroboration, we identified a concurrent increase in PSD95 (and as well as other tripartite synapse proteins including EAAT2 and SV2A) expression. Additionally, the proteomic data obtained from the synaptosomes supported these findings, with the top two GO cellular component terms being neuron to neuron synapses and glutamatergic synapse. Furthermore, we found that M3 increased the number of SA in these mushroom spines. The SA is considered a marker for more mature spines as it is believed to be involved in the storage and release of calcium ions that regulate synaptic transmission and plasticity (Jedlicka et al., 2008; Segal et al., 2010). The strength of synaptic transmission is directly related to the number of SA^+^ in a dendritic spine (Ostroff et al., 2017; Chirillo et al., 2019), and we observed an increase in SA^+^ in both small and large mushroom spines following treatment with M3. In support of this, electrophysiological recordings of basal synaptic output indicated an overall (proportionately) increased synaptic response to equivalent stimuli after M3 treatment. While an increase in SA was not unexpected in large mushroom spines, it was interesting to see an increase in smaller mushroom spines as well. Some studies suggest that SA may serve as a structural scaffold that stabilizes dendritic spines and maintains their shape (Bell et al., 2019; Konietzny et al., 2022). Perhaps the smaller SA^+^ mushrooms represent an early stage of maturation and stabilization mediated by M3 treatment. Overall, M3 strengthens synaptic strength and plasticity putatively by increasing the size and number of SA^+^ mushroom spines which leads to stabilization of the individual mushroom spines cytoarchitecture.

We have previously identified that the PAP is also a primary target site of M3 and demonstrated that M3 increased local protein translation at the PAP, including those involved in glutamate regulation (i.e., EAAT2) and cytoskeletal structure (i.e., actin) (Foster et al., 2018). In our current study, we confirmed that EAAT2 was upregulated in the PAP as expected based on previous experiments with similar pyridazine derivatives. Importantly, we directly observed that M3 increased the number and size of PAPs in contact with mushroom spines. Astrocytes can regulate synaptic plasticity by adjusting the coverage of their processes on synapses (Genoud et al., 2006; Lushnikova et al., 2009; Gavrilov et al., 2018). Larger dendritic spines tend to have more stable interactions with PAPs (Lushnikova et al., 2009). The observed increase in volume of mushroom spines, along with the increased number and size of PAPs in contact with mushroom spines after M3 treatment, indicates that M3 increased both PAP coverage, perhaps as compensation, around the larger mushroom spines. It is not clear, however, whether this change in the PAP structure was due directly to M3 treatment (i.e., before mushroom spine changes) or was a compensatory response (i.e., after mushroom spine changes) to the increased size and function of the mushroom spines. Previous data from a different pyridazine-derivative, interestingly, shows that the proteomic changes to the PAP occur rapidly via local translation (within 4-h of single treatment) (Foster et al., 2018) and therefore suggest that the PAP changes may precede the changes to the mushroom spines.

In summary, this study reveals that a newly developed pyridazine derivative improves synaptic function by enhancing the structure of the tripartite synapse. Since synaptic loss is closely linked to the progression of Alzheimer’s disease, the findings offer additional evidence for M3’s potential therapeutic benefits in AD patients by enhancing synaptic structure and function.

## Data availability statement

The datasets presented in this study can be found in an online repository. The names of the repository and accession number can be found below: PeptideAtlas, PASS05840, http://www.peptideatlas.org/PASS/PASS05840.

## Ethics statement

The animal study was reviewed and approved by the Institutional Animal Care and Use Committee of the Ohio State University.

## Author contributions

ZX: conceptualization, methodology, validation, investigation, formal analysis, data curation, writing–original draft, writing–review and editing, and visualization. JF: conceptualization, methodology, and writing–review and editing. RL, XW, and EB: methodology. GK: methodology and review and editing. C-LL: conceptualization, methodology, resources, writing–original draft, writing–review and editing, visualization, supervision, project administration, and funding acquisition. All authors contributed to the article and approved the submitted version.

## Footnotes

1
https://metascape.org/gp/index.html#/main/step1
